# Collisional dynamics in a gas of molecular super-rotors

**DOI:** 10.1038/ncomms8791

**Published:** 2015-07-10

**Authors:** Yuri Khodorkovsky, Uri Steinitz, Jean-Michel Hartmann, Ilya Sh. Averbukh

**Affiliations:** 1 Department of Chemical Physics, The Weizmann Institute of Science, Rehovot 76100, Israel; 2 Laboratoire Interuniversitaire des Systèmes Atmosphériques (LISA) CNRS (UMR 7583), Université Paris Est Créteil, Université Paris Diderot, Institut Pierre-Simon Laplace, 94010 Créteil, France

## Abstract

Recently, femtosecond laser techniques have been developed that are capable of bringing gas molecules to extremely fast rotation in a very short time, while keeping their translational motion relatively slow. Here we study collisional equilibration dynamics of this new state of molecular gases. We show that the route to equilibrium starts with a metastable ‘gyroscopic stage' in the course of which the molecules maintain their fast rotation and orientation of the angular momentum through many collisions. The inhibited rotational–translational relaxation is characterized by a persistent anisotropy in the molecular angular distribution, and is manifested in the optical birefringence and anisotropic diffusion in the gas. After a certain induction time, the ‘gyroscopic stage' is abruptly terminated by an explosive rotational–translational energy exchange, leading the gas towards the final equilibrium. We illustrate our conclusions by direct molecular dynamics simulation of several gases of linear molecules.

## Abstract (web summary)

Super-rotors are fast rotating molecules whose rotational–translational energy transfer is suppressed. Here the authors study the equilibration of super-rotor gases through molecular dynamics simulations, showing the emergence and explosive termination of an anisotropy in the molecular angular distribution.

## Graphical abstract

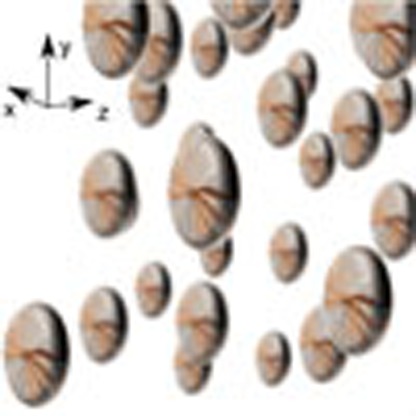

The efficiency of the collisional energy transfer from the internal molecular degrees of freedom (vibrational or rotational) to the translational ones is crucially dependent on the typical timescales of the processes involved. To analyse the rotational–translational (RT) energy transfer between colliding molecules in a gas, one has to compare the typical rotational period, *t*_rot_=2*π*/*ω*_rot_ (*ω*_rot_ is the angular rotational frequency) and the characteristic duration of a collision, *t*_coll_. The latter may be estimated as the time it takes for a molecule of the typical speed *v* to traverse the range *σ* of the intermolecular potential: *t*_coll_∼2*σ*/*v*. The ratio of these two timescales is the so-called adiabaticity parameter *a*=*t*_coll_/*t*_rot_. When *t*_coll_ is long enough for the molecule to complete several revolutions during the collision, that is, *a*≫1, the collision is adiabatic with respect to the rotational dynamics of the molecule. During the encounter of two molecules, the molecular rotational dynamics adiabatically adjusts itself to a slowly varying intermolecular interaction, and the rotational energy of the molecules returns to its initial value after the end of the collision. In such cases, the RT relaxation is suppressed, and the fast-rotating molecules are dubbed super-rotors (SRs)^1^. On the other hand, when *a*∼1 (or smaller), the collisions are impulsive, and efficient energy transfer occurs between the rotational and the translational degrees of freedom. Similar arguments are relevant also to the vibrational–translational relaxation process, and they are known since the introduction of the Landau–Teller theory^2^ describing the vibrational–translational energy exchange in a collision between an atom and a diatomic molecule. The main difference between rotations and vibrations is the much smaller typical timescale of the latter, which makes the ‘adiabatic regime' more accessible for the vibrations. In addition, the dependence of rotational frequency on energy provides a threshold for adiabaticity. It has been known for a while that highly rotationally excited molecules appearing in some exothermic chemical reactions and photodissociation processes may exhibit extended collisional relaxation^3^. More recently, it has been predicted that trapped ultra-cold molecules may also experience adiabatic suppression of the rotational relaxation^1^,^4^, which may open new options for cold chemistry (see ref. 5 and references therein). In the latter case the translational temperatures are very low, and the adiabaticity is enhanced by the prolonged duration of the collisions that are quantum in nature. Nowadays, molecules can be excited to become SRs by optical techniques aiming at inducing molecular alignment by short laser pulses (for reviews see refs 6, 7, 8), especially utilizing the schemes using multiple laser pulses^9^. Moreover, several methods have been suggested and demonstrated for converting the transient molecular alignment into a concerted unidirectional molecular rotation, including the techniques of ‘optical centrifuge'^10^,^11^,^12^,^13^,^14^, ‘molecular propeller'^15^,^16^,^17^ and ‘chiral train' of laser pulses^18^.

Our research is stimulated by recent laser experiments that achieved the SR regime at near-ambient conditions^9^,^12^,^13^,^14^. The optical centrifuge method was recently used^19^ to investigate the effects of ultrafast molecular rotation on the initial short-lasting relaxation stage of collisional decoherence of the quantum Raman transitions in molecular nitrogen. At ambient conditions, this stage lasts <1 ns (ref. 19), after which coherent quantum effects die completely and become irrelevant. At the same time, optical centrifuge experiments^12^,^13^ demonstrated nontrivial non-equilibrium collisional kinetics in a gas of SRs, which lasted orders of magnitude longer than the quantum decoherence processes studied in ref. 19. The theoretical analysis of these kinetic phenomena is still in its infancy, and the previous quantum treatments focused on ultra-cold collisions of SRs^4^,^5^ are intractable in this case.

Here, we investigate the collisional dynamics of the RT energy transfer and collisional reorientation in a gas of molecules being initially at room temperature, but with extremely high rotational excitation. We examine collisional relaxation of SRs in an extended time range, starting from a highly non-equilibrium initial state till the complete thermalization. Our study provides the first modelling (to the best of our knowledge) of the collisional kinetics of molecular SRs by direct molecular dynamics (MD) simulation methods. The classical approach seems to be an appropriate tool for studying molecules at room temperature, excited to the quasi-classical rotational states with the angular momentum of tens to hundreds of ℏ. It has already been successfully used for analysis of the laser experiments on molecular alignment at dissipative conditions^20^,^21^,^22^. We studied the relaxation dynamics for several small linear molecules (such as N_2_, O_2_ and CO_2_) and found that they demonstrate a rather universal behaviour in the SR regime. Therefore, we present below in detail our results for the representative case of molecular nitrogen, ^14^N_2_. The Supplementary Movie 1 shows in an animated way a very similar relaxation dynamics in a gas of O_2_ molecules.

## Results

### Energy relaxation

**Figure 1:**
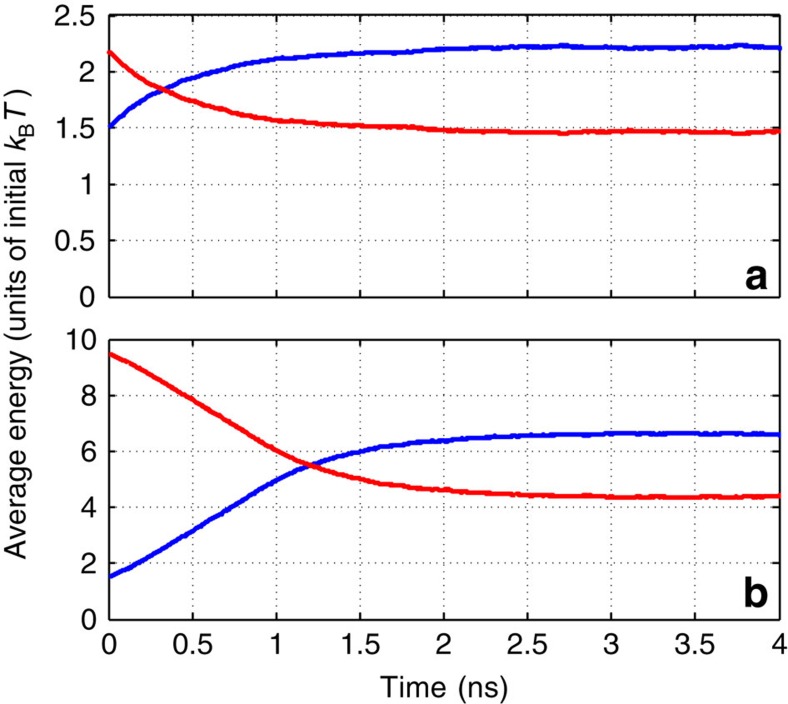
Exponential energy relaxation. Equilibration of energy with time for a gas of 9,261 ^14^N_2_ molecules being initially at ambient temperature and pressure, and kicked by a linearly polarized laser pulse with a kick strength of (**a**) *P*=15 and (**b**) *P*=40. The mean translational and rotational energy of the molecules are plotted in blue and red, respectively.

We start by presenting results of a test case of a moderate rotational excitation of molecules by a single non-resonant short laser pulse having linear polarization. Such a pulse is quantified by the so-called ‘kick strength' parameter, *P*, that defines a typical amount of the angular momentum (in units of ℏ) transferred from the laser pulse to a molecule (see the Methods section). The simulation of the initial rotational state by laser excitation was performed as in ref. 17. Figure 1a demonstrates an exponential decay of the excess rotational energy of N_2_ molecules after the pulse of *P*=15, and the corresponding increase of the translational energy as a function of time. Such a kick strength (and even higher) can be achieved^9^ by a train of several laser pulses (50 fs duration, 800 nm central wavelength and peak intensity of 36 TW cm^−2^) separated in time by the rotational revival time ∼8.4 ps of ^14^N_2_ molecules. The magnitude of this kick is comparable to the thermal value of the angular momentum quantum number *J*∼10 for nitrogen molecules at *T*=300 K. At the end of the relaxation process, the ratio between the average translational and rotational energies is 3/2, as required by the equipartition theorem. The gas reaches equilibrium after about 2 ns (after each molecule has experienced about 20 collisions). Such an exponential relaxation pattern is in agreement with other recent theoretical^20^,^23^ and experimental^21^,^22^,^24^ studies on the collisional relaxation of laser-excited molecular rotations. For a stronger laser pulse of *P*=40, a slight deviation from the exponential behaviour appears at short times, see Fig. 1b.

**Figure 2:**
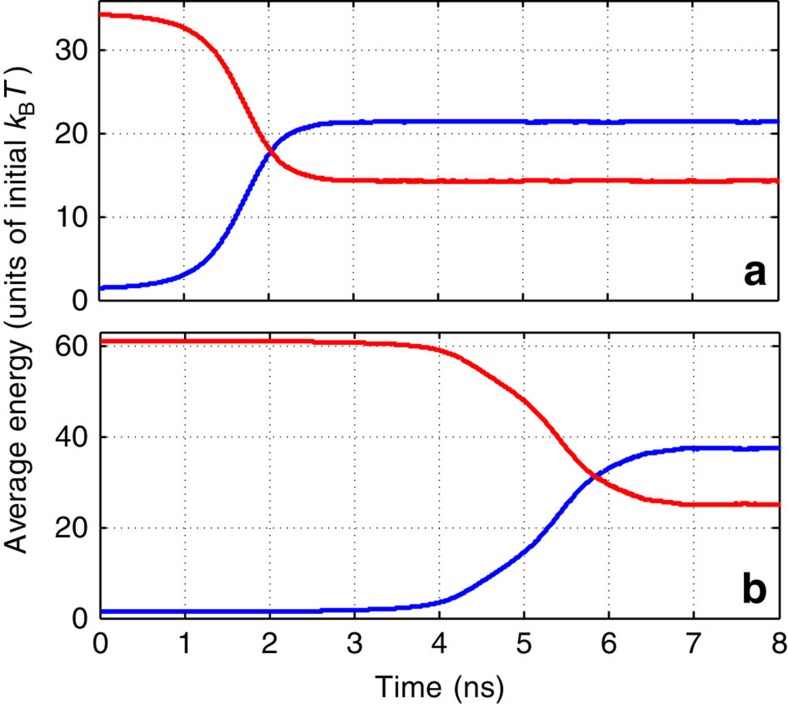
Explosive energy relaxation. Equilibration of energy with time for a gas of ^14^N_2_ molecules being at ambient temperature and pressure, and subject to an optical centrifuge at *t*=0. (**a**,**b**) Initial angular momentum of *J*=60 and 80, respectively. The mean translational and rotational energies of the molecules are plotted in blue and red, respectively.

We now demonstrate that the relaxation scenario is drastically different for higher-rotational states that are attainable by the ‘optical centrifuge' technique^10^,^11^,^12^,^13^,^14^. Figure 2 presents the results of MD simulations for an ensemble of nitrogen molecules that were all initially aligned along the *x* axis and brought to fast rotation in the *xy* plane with the same initial angular momentum ***J*** oriented along the *z* axis. This initial condition relates to molecules suddenly released from an ideal optical centrifuge. Despite its simplicity, it satisfactory describes the molecules released from a real centrifuge, as shown in our forthcoming publication (Y. Khodorkovsky, U. Steinitz, J.-M. Hartmann and I.Sh. Averbukh, manuscript in preparation) that investigates in detail the centrifugation process using excitation parameters from experimental papers^12^,^13^,^14^ (centrifuge pulses about 50–100 ps long, their linear polarization undergoing an accelerated rotation and reaching the angular frequency of 10 THz by the end of the pulse). In this article, we concentrate on the novel relaxation phenomena in a gas of SRs, and use the simplest description of the initial preparation stage. Figure 2 displays the RT energy transfer dynamics for molecules initially excited to *J*=60, and 80. It was obtained by averaging the results of multiple runs of the MD simulations involving ∼10^4^ molecules. Initially, for a relatively long period covering several tens of collisions, there is almost no change in the mean rotational energy of the molecules (plateau region). After a certain time, the plateau is followed by an abrupt, explosive-like energy dump from the rotational motion into the translational one, leading to the final thermal equilibration of the heated gas.

**Figure 3:**
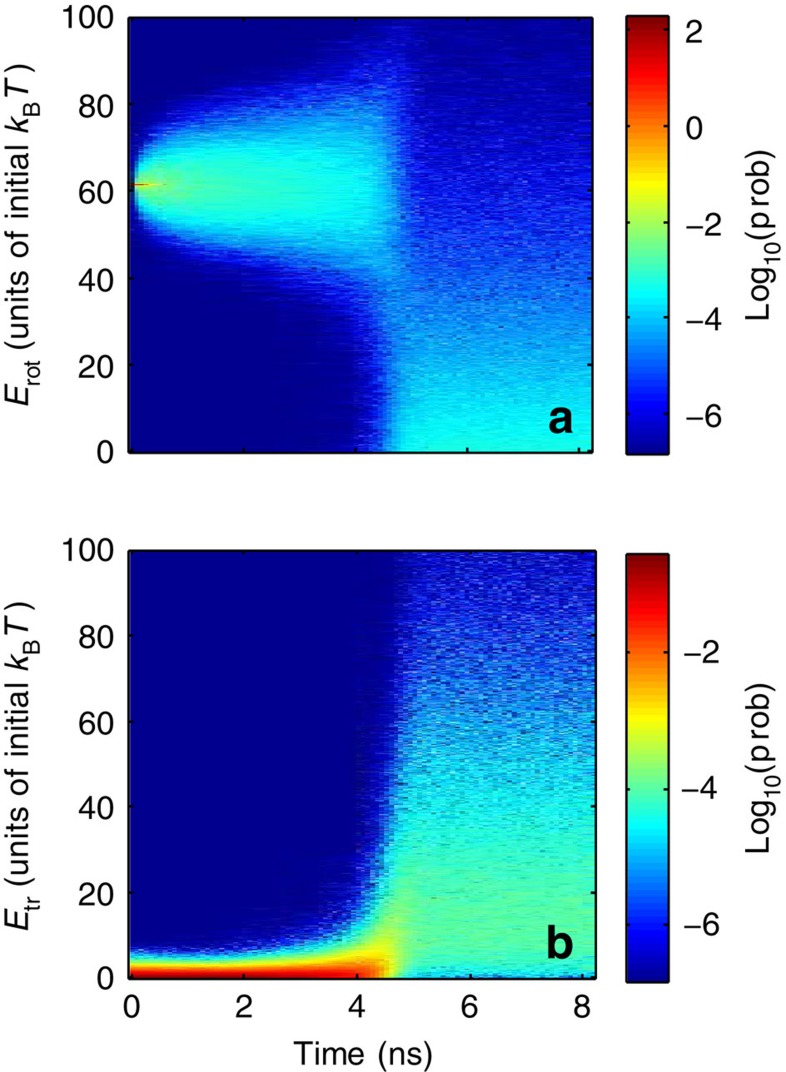
Time-dependent distributions. Density plots for time-dependent distributions of the (**a**) rotational and (**b**) translational energies. The molecules are initially centrifuged to *J*=80. An explosive transition towards the thermal Maxwell-Boltzmann distribution of the heated gas is clearly seen near 5 ns (compare with Fig. 2b).

A deeper insight is given by the numerically simulated time-dependent distribution functions for the rotational (Fig. 3a) and translational (Fig. 3b) energies. They show that during the plateau period, the rotational distribution broadens mainly due to the rotation–rotation energy exchange, and both distributions markedly change their shapes in the course of the explosive relaxation. There is a clear physical reason for such relaxation dynamics. During the plateau period, the RT relaxation is inhibited due to the high value of the adiabaticity parameter, *a*≫1. However, as the rotational distribution broadens, and the energy leaks from rotations to translations, there is an increasing fraction of molecules colliding at lower values of *a*. This makes the RT relaxation more efficient and triggers a positive-feedback mechanism. The increase in the translational speeds shortens the typical collision time and raises the heating rate of the gas, which in turn accelerates further the RT energy transfer and ignites an explosive thermalization.

The duration of the plateau period grows fast with the increase of the initial rotational excitation and/or the decrease of the initial translational temperature, due to the rise of the typical adiabaticity parameter *a*. On the contrary, the duration of the explosive RT energy exchange experiences much smaller variations with these two parameters. Statistical analysis of multiple MD simulations shows that the actual shape of the transition stage seen at Fig. 2 is determined both by the dynamics of individual explosion events and the distribution function of randomly distributed explosion times. The mechanism of explosive relaxation has much in common with the famous phenomenon of thermal explosion^25^ in heat-generating solids, which was first considered by N. Semenov in 1928. We provide a detailed and elaborated discussion of the explosive relaxation of SRs in terms of Semenov's theory of branched-chain reactions in our forthcoming publication (Y. Khodorkovsky, U. Steinitz, J.-M. Hartmann and I.Sh. Averbukh, manuscript in preparation).

We performed our MD simulations at several initial temperatures, and observed that the explosive kinetics is more emphasized at lower temperatures. This is naturally expected as the adiabaticity parameter *a* increases when lowering the translational temperature. We provide the representative results at room temperature, as such conditions are typical to the current experiments on molecular SRs^12^,^13^,^14^.

The Supplementary Movie 1 presents in an animated way the relaxation scenario in the gas of oxygen SRs. It shows the same generic relaxation stages (inhibited and explosive) as in the above example of the nitrogen gas, and demonstrates that the relaxation dynamics is rather insensitive to the specific choice of molecules, or an exact form of the intermolecular potential. A shorter timescale is attributed to the higher gas pressure chosen for that numerical example.

### Molecular reorientation

**Figure 4:**
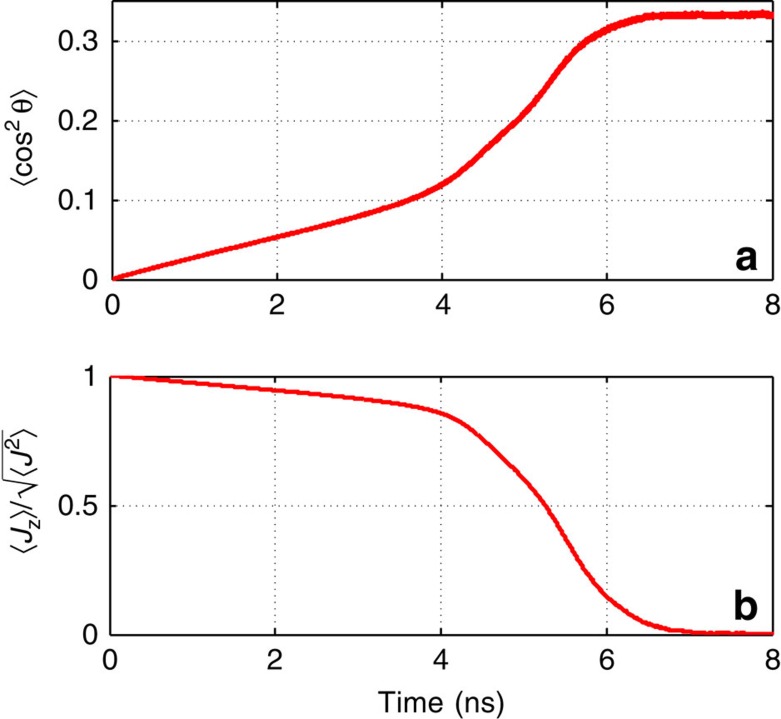
Molecular reorientation. Orientation dynamics of the molecules that are initially centrifuged in the *xy* plane, *J*=80. (**a**) Time dependence of the alignment factor along the *z* axis. (**b**) Decay of the normalized *z*-component of the angular momentum.

To get a deeper insight into the collisional relaxation of SRs, we investigated the process of isotropization of the molecular orientation, and of the direction of the rotational angular momentum. Figure 4a shows that during the plateau period (until about 4 ns), the factor of alignment along the *z* axis, 〈cos^2^*θ*〉, remains close to zero (here *θ* is the angle between the molecular axis and *z* axis). This means that the molecules continue rotating mainly in the *xy* plane, with the angular momentum vector oriented along the *z* axis. This is also supported by Fig. 4b that shows the time dependence of the *z*-component (parallel to the exciting laser's propagation direction) of the molecular angular momentum, *J*_*z*_. The ability to keep orientation of the angular momentum is characteristic of gyroscopes (even as small as a rotating molecule). Such a tendency is consistent with a much weaker propensity observed in recent studies of laser-excited molecules^20^ at lower degrees of rotational excitation. For this reason, we term the whole period before the self-accelerating RT energy exchange as a ‘gyroscopic stage' of relaxation. As seen from Fig. 4, the isotropization of the SRs occurs mainly during the stage of the explosive relaxation, after which the alignment factor takes the isotropic value of 〈cos^2^*θ*〉=1/3, and there is no preferred orientation of the angular momentum vector.

**Figure 5:**
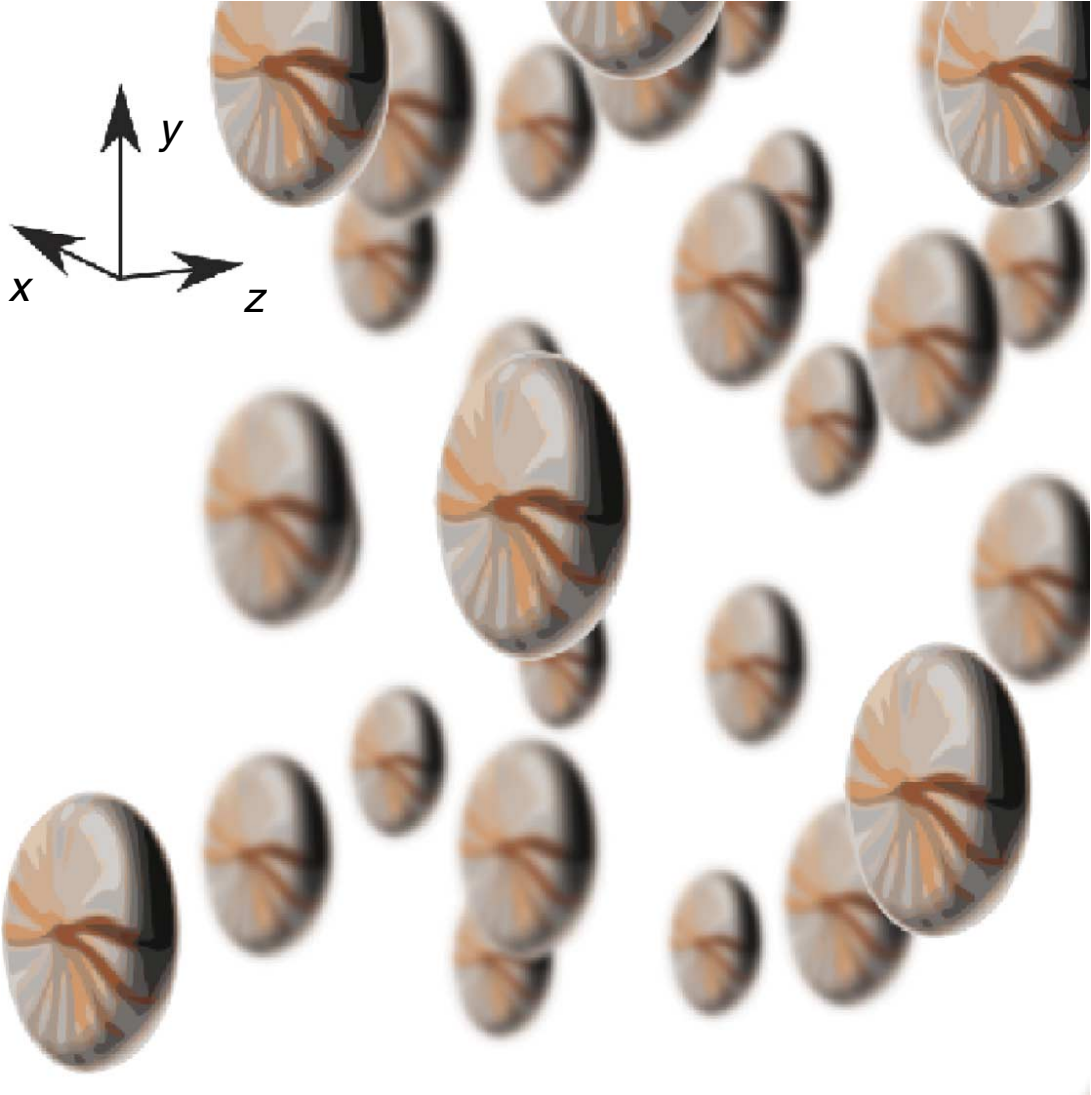
‘Pancake'-shape distribution. Schematic illustration of the anisotropic angular distribution of gas molecules spun by an optical centrifuge rotating in the *xy* plane.

Figure 5 schematically shows the anisotropic (pancake-like) angular distribution maintained by the molecular SRs in the course of many collisions during the gyroscopic stage. The initial fast rotation was set by an optical centrifuge operating in the *xy* plane. The macroscopic polarizability of such a gas is anisotropic as well, which can be detected by measuring birefringence for a polarized light propagating along any direction in the *xy* plane. Such a persistent birefringence may serve as an optical indicator of the gyroscopic relaxation stage. However, here we will focus on the gas-kinetics manifestations of this anisotropy. Just looking at Fig. 5, one may expect that the collisional cross-section for the face-to-face colliding molecular ‘pancakes' should be different from the cross-section for the side-to-side collisions. Also, the RT energy exchange happens differently in the cases of the face-to-face and side-to-side collisions^13^. This suggests the emergence of anisotropic transport phenomena in the gas, such as anisotropic mass and energy diffusion. Moreover, the explosive RT energy exchange at the end of the gyroscopic phase should have its manifestations in the transport effects as well.

### Accelerating and anisotropic diffusion

**Figure 6:**
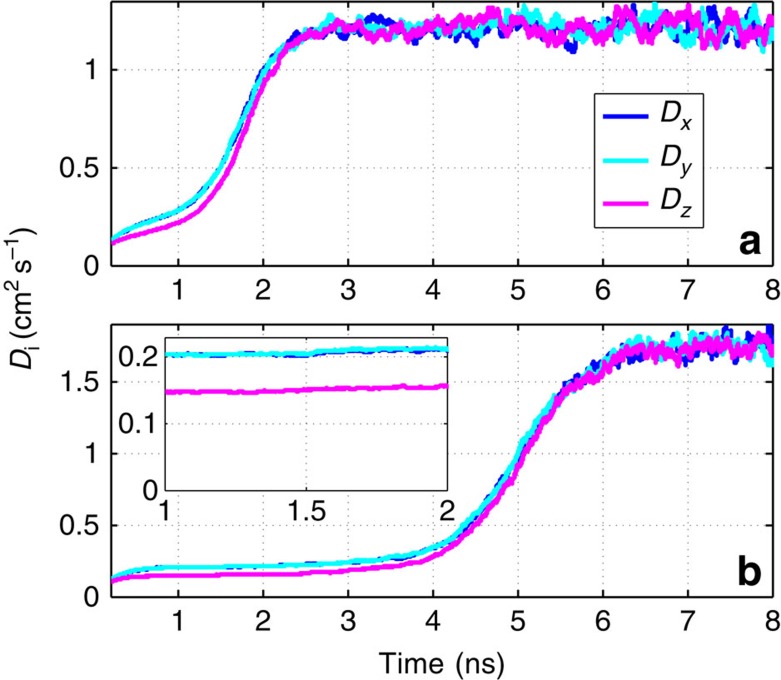
Accelerating and anisotropic diffusion. Time-dependent diffusion coefficients *D*_*i*_(*t*) along the axes *i*= *x*,*y*,*z* are plotted as a function of time. The molecules in **a** and **b** are initially centrifuged in the *xy* plane with an angular momentum of *J*=60 and *J*=80, respectively. Inset of **b** is an enlarged view of **b** from 1 to 2 ns.

To test these predictions, we investigated the process of self-diffusion of a nitrogen molecular SR by means of the above direct MD simulations. Following the random-walk trajectory for each molecule of an ensemble prepared in a centrifuged state, we calculated the average square of the cumulative molecular displacement along each of the Cartesian axes as a function of time. The time-dependent diffusion coefficients for the motion along every axis was derived by differentiating this function with respect to time (for example, 
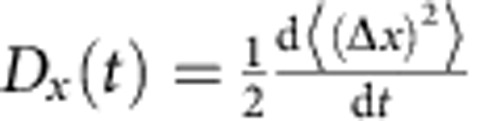
). Figure 6b shows that as long as the molecules keep the original orientation of their rotation axis (gyroscopic stage), the diffusion coefficients in the *x*- and *y*-directions are indeed about 40% larger than the one corresponding to the diffusion along the *z* axis. The diffusion coefficients grow with time (as seen in Fig. 6), because of the gradual heating of the translational motion of the molecules due to the RT energy exchange. With the ignition of the explosive relaxation, the diffusion coefficients grow abruptly together with the translational speeds, and all three coefficients reach the same high value corresponding to the final hot equilibrated state.

## Discussion

Nontrivial regimes of mass and heat transfer in the gas of SRs may happen when the laser excitation takes place in a spatially confined domain, like the focal spot of a focused laser beam, which implies that the ‘cloud' of centrifuged molecules disperses differently in the axial and radial directions. The explosive-like delivery of the rotational energy to the translational motion leads to an increased local pressure and to the subsequent generation of sound waves, as well as to the creation of a hot area with a reduced molecular concentration, which may be optically observed^26^,^27^,^28^. This density hole is then slowly healed via the diffusion of cold unexcited molecules from the area outside the focal spot, while the hot molecules diffuse outwards. The multi-scale long decay of rotational signals from the optically centrifuged molecules in dense gases reported recently^12^,^13^ may be actually related to the above interplay of the kinetic and hydrodynamic phenomena, which requires additional theoretical analysis of these experiments.

Finally, we emphasize that although the molecular gas arrives to a microscopically thermalized state, macroscopic non-equilibrium flows can still exist in the gas. For molecules that are initially excited into a rotation with a preferred sense of spinning (with oriented mean angular momentum), it was shown recently that a vortex flow appears around the laser focal spot after thermalization^28^.

To summarize, we investigated theoretically the collisional relaxation in a gas of molecular SRs, excited with the help of ultrashort laser pulses. We found that the SRs behave like tiny gyroscopes after the excitation, and maintain their fast rotation and orientation of the angular momentum through many collisions. This leads to the anisotropic diffusion in the gas, and to a long-lasting optical birefringence. The RT energy transfer is effectively suppressed during the gyroscopic stage. This metastable state of the gas terminates abruptly after some induction period (depending on the initial rotational excitation), due to development of an explosive self-accelerating energy transfer from the rotational degrees of freedom to the translational ones. We discussed optical means for detecting the macroscopic effects associated with SRs relaxation. Other manifestations may include filamentation effects, anisotropic diffusion of selected species in a molecular mixture of different components, anisotropic viscosity in the gas and directed acousto-optical signals produced by the laser-generated rotationally excited gas^29^.

## Methods

### Initial conditions

The simulations were carried out with 9,261 molecules residing in a cubic volume at room translational temperature and pressure. Initially, the molecules were uniformly distributed in space, while their initial velocity followed the Maxwell–Boltzmann distribution. The initial distribution of the rotational degrees of freedom (direction of molecular axes, angular velocity vectors) was obtained by simulating the process of ultrafast laser excitation.

### Rotational excitation

In the case of the excitation by a linearly polarized aligning laser pulse (see Fig. 1), the molecules were impulsively excited as if affected by a linearly polarized femtosecond laser pulse with a kick strength 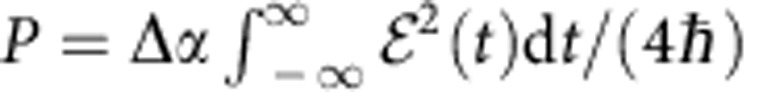 (where Δ*α* is the molecular polarizability anisotropy and 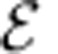 is the envelope of the electric field of the laser pulse), as described in detail in ref. 17. Parameter *P* defines a typical amount of the angular momentum (in units of ℏ) transferred from the laser pulse to a molecule. To explore the relaxation dynamics of the centrifuged molecules (Figs 2, 3, 4 and 6), we considered ensembles of molecules that were initially fast rotating in some plane with a well-defined angular speed, or with a narrow Gaussian distribution of the rotational speeds, and investigated the dependence of the kinetics on the initial *J*-value. The tested angular speeds were in the range of experimental data reported in refs 12, 13, 14.

### Time evolution

Starting from these initial conditions, we let the molecules propagate in space using the standard Verlet algorithm, and periodic boundary conditions, while the propagation of the rotational motion was done as described in ref. 30. The classical intermolecular interaction potential was taken from ref. 31.

## Additional information

**How to cite this article:** Khodorkovsky, Y. *et al*. Collisional dynamics in a gas of molecular super-rotors. *Nat. Commun.* 6:7791 doi: 10.1038/ncomms8791 (2015).

## Supplementary Material

Supplementary Movie 1 Animated visualization of the collisional dynamics in a gas of oxygen super-rotors. This video depicts the simulation of ~200 spinning oxygen molecules, initially at room translational temperature, all rotating fast at J~60. For visualization purposes the initial concentration is 20 times that of atmospheric gas and the boundary conditions are periodic. The resulting dynamics follows the same generic scenario as observed in the molecular dynamics simulations for nitrogen molecules in the main text of the paper, only faster by a factor of about 20 (due to the increased pressure). In the left box each particle is represented by a spindle whose axis corresponds to the rotation axis, and its length depicts the rotation speed. The color of each particle codes the translational speed. On the right panel, the evolution of the mean translational and rotational energy is shown (compare with Fig. 2 in the main text), along with the momentary distribution of the rotational energy (green markers). The first quarter of the movie features the metastable gyroscopic stage, where the molecules' rotation axes are still parallel to the original preferred direction, despite the many collisions each of them has undergone. The stage is followed by the explosive relaxation (at around halfway through the movie) where the molecules suddenly transform from slow (blue) parallel spindles into fast moving (red) ones with slowed, isotropic rotation.

## References

[b1] Li J., Bahns J. T. & Stwalley W. C. Scheme for state-selective formation of highly rotationally excited diatomic molecules. J. Chem. Phys. 112, 6255–6261 (2000).

[b2] Landau L. & Teller E. On the theory of sound dispersion. Phys. Z. Sow. 10, 34–43 (1936).

[b3] Polanyi J. C. & Woodall K. B. Mechanism of rotational relaxation. J. Chem. Phys. 56, 1563–1572 (1972).

[b4] Forrey R. C. Cooling and trapping of molecules in highly excited rotational states. Phys. Rev. A 63, 051403(R) (2001).

[b5] al-Qady W. H., Forrey R. C., Yang B. H., Stancil P. C. & Balakrishnan N. Cold collisions of highly rotationally excited CO2 with He: the prospects for cold chemistry with super-rotors. Phys. Rev. A 84, 054701 (2011).

[b6] Stapelfeldt H. & Seideman T. Colloquium: aligning molecules with strong laser pulses. Rev. Mod. Phys. 75, 543–557 (2003).

[b7] Ohshima Y. & Hasegawa H. Coherent rotational excitation by intense nonresonant laser fields. Int. Rev. Phys. Chem. 29, 619–663 (2010). 10.1021/jp102840t 20961157

[b8] Fleischer S., Khodorkovsky Y., Gershnabel E., Prior Y. & Averbukh I. Sh. Molecular alignment induced by ultrashort laser pulses and its impact on molecular motion. Isr. J. Chem. 52, 414–437 (2012).

[b9] Cryan J. P., Bucksbaum P. H. & Coffee R. N. Field-free alignment in repetitively kicked nitrogen gas. Phys. Rev. A 80, 063412 (2009).

[b10] Karczmarek J., Wright J., Corkum P. & Ivanov M. Optical centrifuge for molecules. Phys. Rev. Lett. 82, 3420–3423 (1999).

[b11] Villeneuve D. M. . Forced molecular rotation in an optical centrifuge. Phys. Rev. Lett. 85, 542–545 (2000). 10991335 10.1103/PhysRevLett.85.542

[b12] Yuan L., Teitelbaum S. W., Robinson A. & Mullin A. S. Dynamics of molecules in extreme rotational states. Proc. Natl Acad. Sci. USA 108, 6872–6877 (2011).

[b13] Toro C., Liu Q., Echebiri G. O. & Mullin A. S. Inhibited rotational quenching in oriented ultra-high rotational states of *CO*_2_. Mol. Phys. 111, 1892–1901 (2013).

[b14] Korobenko A., Milner A. A. & Milner V. Direct observation, study, and control of molecular super rotors. Phys. Rev. Lett. 112, 113004 (2014). 24702361 10.1103/PhysRevLett.112.113004

[b15] Fleischer S., Khodorkovsky Y., Prior Y. & Averbukh I. Sh. Controlling the sense of molecular rotation. New J. Phys. 11, 105039 (2009).

[b16] Kitano K., Hasegawa H. & Ohshima Y. Ultrafast angular momentum orientation by linearly polarized laser fields. Phys. Rev. Lett. 103, 223002 (2009). 20366091 10.1103/PhysRevLett.103.223002

[b17] Khodorkovsky Y., Kitano K., Hasegawa H., Ohshima Y. & Averbukh I. Sh. Controlling the sense of molecular rotation: classical versus quantum analysis. Phys. Rev. A 83, 023423 (2011).

[b18] Zhdanovich S. . Control of molecular rotation with a chiral train of ultrashort pulses. Phys. Rev. Lett. 107, 243004 (2011). 22242996 10.1103/PhysRevLett.107.243004

[b19] Milner A. A., Korobenko A., Hepburn J. W. & Milner V. Effects of ultrafast molecular rotation on collisional decoherence. Phys. Rev. Lett. 113, 043005 (2014). 25105617 10.1103/PhysRevLett.113.043005

[b20] Hartmann J.-M. & Boulet C. Quantum and classical approaches for rotational relaxation and nonresonant laser alignment of linear molecules: a comparison for *CO*_2_ gas in the nonadiabatic regime. J. Chem. Phys. 136, 184302 (2012). 22583282 10.1063/1.4705264

[b21] Vieillard Th. . Field-free molecular alignment for probing collisional relaxation dynamics. Phys. Rev. A 87, 023409 (2013).

[b22] Karras G. . Using molecular alignment to track ultrafast collisional relaxation. Phys. Rev. A 89, 063411 (2014).

[b23] Ramakrishna S. & Seideman T. Intense laser alignment in dissipative media as a route to solvent dynamics. Phys. Rev. Lett. 95, 113001 (2005). 16196999 10.1103/PhysRevLett.95.113001

[b24] Owschimikow N. . Cross sections for rotational decoherence of perturbed nitrogen measured via decay of laser-induced alignment. J. Chem. Phys. 133, 044311 (2010). 20687654 10.1063/1.3464487

[b25] Semenov N. N. On the theory of combustion processes. Z. Phys. Chem. 48, 571–582 (1928).

[b26] Zahedpour S., Wahlstrand J. K. & Milchberg H. M. Quantum control of molecular gas hydrodynamics. Phys. Rev. Lett. 112, 143601 (2014). 24765959 10.1103/PhysRevLett.112.143601

[b27] Lahav O. . Long-lived waveguides and sound-wave generation by laser filamentation. Phys. Rev. A 112, 021801(R) (2014).

[b28] Steinitz U., Prior Y. & Averbukh I. Sh. Laser-induced gas vortices. Phys. Rev. Lett. 109, 033001 (2012). 22861845 10.1103/PhysRevLett.109.033001

[b29] Schippers W. . Stimulated Raman rotational photoacoustic spectroscopy using a quartz tuning fork and femtosecond excitation. Appl. Phys. B 105, 203–211 (2011).

[b30] Allen M. P. & Tildesley D. J. Computer Simulation of Liquids Oxford Univ. Press (1989).

[b31] Cheung P. S. Y. & Powles J. G. The properties of liquid nitrogen. IV. A computer simulation. Mol. Phys. 30, 921–949 (1975).

